# Hair Follicle Morphogenesis in the Treatment of Mouse Full-Thickness Skin Defects Using Composite Human Acellular Amniotic Membrane and Adipose Derived Mesenchymal Stem Cells

**DOI:** 10.1155/2016/8281235

**Published:** 2016-08-15

**Authors:** Wu Minjuan, Xiong Jun, Shao Shiyun, Xu Sha, Ni Haitao, Wang Yue, Ji Kaihong

**Affiliations:** Department of Histology and Embryology, Second Military Medical University, Shanghai 200433, China

## Abstract

Early repair of skin injury and maximal restoration of the function and appearance have become important targets of clinical treatment. In the present study, we observed the healing process of skin defects in nude mice and structural characteristics of the new skin after transplantation of isolated and cultured adipose derived mesenchymal stem cells (ADMSCs) onto the human acellular amniotic membrane (AAM). The result showed that ADMSCs were closely attached to the surface of AAM and grew well 24 h after seeding. Comparison of the wound healing rate at days 7, 14, and 28 after transplantation showed that ADMSCs seeded on AAM facilitated the healing of full-thickness skin wounds more effectively as compared with either hAM or AAM alone, indicating that ADMSCs participated in skin regeneration. More importantly, we noticed a phenomenon of hair follicle development during the process of skin repair. Composite ADMSCs and AAM not only promoted the healing of the mouse full-thickness defects but also facilitated generation of the appendages of the affected skin, thus promoting restoration of the skin function. Our results provide a new possible therapy idea for the treatment of skin wounds with respect to both anatomical regeneration and functional restoration.

## 1. Introduction

Local or systemic cutaneous lesions arising from skin injury are often related to the loss of barrier function. Early repair of skin injury and maximal restoration of the function and appearance have become important targets of clinical treatment. Autologous free skin grafting, skin flap transplantation, and allogenous or xenogeneic skin transplantation remain the first consideration in conventional clinical treatment of skin injury [1, 2]. Although these techniques are usually effective in most cases, how to solve the problem of covering large wound areas and reduce wound retraction and scar formation in patients with large and life-threatening wounds or those with beauty demands remains a clinical challenge. Construction of an ideal skin substitute has become an inevitable trend in burn and plastic surgery. In 1975, Rheinwald and Green [3] first reported successful treatment of wounds with transplantation of cultured human epidermal cells, which symbolizes a milestone in wound treatment. With the development of modern molecular and cellular biology and tissue engineering, advances in skin substitute research and application have gradually rendered it possible to reduce secondary injury from autologous skin transplantation [4]. Subsequently, researchers have created techniques of autologous epidermal cell culture and transplantation for the treatment of burn and various other acute/chronic wounds, thus providing permanent coverage for large-area wounds. However, the anti-infection ability of these skin substitutes is relatively low, and their functional and appearance degradation is also an unavoidable problem.

Epidermal substitutes are mainly used for superficial wounds [5]. The epidermis alone cannot survive long for large, deep, and extensive wounds because it cannot receive nutritional support from the dermis and therefore needs mechanical protection of a dermal substitute. The dermal composition in the skin substitute can prevent the wound from retracting and increase mechanical stability. Knowing that the dermis plays an important role in the regulation of epidermal renewal and reconstruction, accelerating the construction of the dermis is an extremely important link in skin tissue engineering [6].

The human amniotic membrane (hAM) is a natural high-molecular biological material and can express multiple growth factors and mRNA-related proteins including collagen, glycoprotein, protein polysaccharide, integrin, and lamellar body, which are beneficial to cell growth and reproduction. For this reason, hAM is often used as a vector for cell growth and proliferation [7, 8]. Acellular amniotic membrane (AAM) is a natural biologic scaffold and can be used as an extracellular matrix to load cells for the construction of engineered tissues and organs [9]. There have been many reports about the use of AAM for wound coverage [10, 11]. But few studies have reported the use of composite AAM and stem cells for the treatment of skin defects and functional repair. In the present study, we intended to observe the healing of skin defects and histological and structural characteristics of the newborn skin after transplantation of isolated and cultured adipose derived mesenchymal stem cells (ADMSCs) onto AAM and using them to cover the skin defects in nude mice, in an attempt to explore the possibility of seeding ADMSCs on AAM to repair skin defects.

## 2. Materials and Methods

### 2.1. Characterization of ADMSCs

Fourth-passage ADMSCs stored in our laboratory were characterized for the expression pattern of mesenchymal and pluripotent markers by immunohistochemistry and flow cytometry. P4 ADMSCs were fixed with 4% paraformaldehyde in phosphate buffer for 4 min at room temperature. After being blocked with PBS containing 2% BSA, cells were permeabilized with 0.1% Triton-X 100 for 10 min. Slides were incubated sequentially overnight at 4°C with the following primary antibodies: Oct-4 (goat polyclonal, 1 : 50, Santa Cruz Biotechnology Inc.) and SH-2 (1 : 50, mouse polyclonal, Santa Cruz Biotechnology Inc.). The slides were washed with PBS ± 1% BSA after each step. Finally, cells were incubated for 40 min at room temperature with FITC or Cy3-coupled anti-goat or anti-mouse IgG secondary antibody (1 : 500, Jackson) and observed under a fluorescence microscope (BX41TB, Olympus, Tokyo, Japan).

For FACS analysis, cells were trypsinized and spun down by centrifugation for 5 min at 1000 rpm. The cell pellet was resuspended in 100 *μ*L PBS and incubated on ice for 30 min with FITC or PE-conjugated monoclonal antibodies against CD29, CD44, CD105, CD90, CD34, and CD45 (Becton-Dickinson, San Jose, CA). After two washes with cold PBS, the labeled cells were analyzed with a FACStar flow cytometer (Becton-Dickinson, San Jose, CA, USA).

### 2.2. Preparation and Evaluation of AAM

hAM was decellularized using 0.03% (w/v) sodium dodecyl sulfate (SDS), with hypotonic tris buffer and protease inhibitors and nuclease treatment. Both intact hAM and AAM were fixed in 4% paraformaldehyde before HE staining to observe whether the epidermal layer of AAM had been removed completely.

#### 2.2.1. Seeding of ADMSCs on AAM

P4 adipose stem cells were seeded on the surface of AAM at a density of 2 × 10^5^ cells/cm^2^, with the KC-SFM medium replaced on alternative days. After 7-day loading, cell adhesion and growth were observed under an optical microscope by HE staining.

### 2.3. Preparation of Full-Thickness Skin Defects in Nude Mice and Repair of the Skin Defects in Different Groups

After intraperitoneal anesthesia, full-thickness skin defects were made by cutting a 0.8 cm × 0.8 cm wound on the back deep to the fascia in 6-week-old healthy BALB/C-nu mice of either sex weighing 20~30 g. According to the different methods used for wound treatment, the 30 BALB/C-nu mice were equally randomized to three groups: hAM group, where the wounds were treated by native hAM; AAM group, where the wounds were treated with AAM alone; and ADMSCs seeded on AAM group, where the wounds were treated by transplanting composite ADMSCs and AAM to the wounds. Before treatment, the wounds were gently rinsed with gentamycin normal saline (NS). AAM was fixed on the surrounding skin with 1.0-gauge suture and dressed with NS gauze.

### 2.4. Observation and Measurement

Wound contraction: the wound area was measured 7, 14, and 28 days after transplantation in all groups, and the wound healing rate was calculated using the following equation: would healing rate = (original wound area − current wound area)/original wound area × 100%.

### 2.5. Immunohistofluorescence Analysis and HE Staining Observation

Immunohistofluorescence staining was used to examine the expression of cytokeratin 19 (CK19) and human derived mitochondria in the new skin tissue. At day 28 after treatment, OCT-embedded specimens were made into 10 *μ*m thick continuous frozen sections. After being fixed with cold acetone, the slides were preincubated in the working normal goat serum concentration for 10 min and then incubated with a mouse polyclonal anti-CK19-specific antibody (1 : 50, Dako) or human derived mitochondria (1 : 200, Neomarks) at 4°C for 16 h. Then, the slides were incubated for 40 min at room temperature with FITC or Cy3-coupled IgG secondary antibody (1 : 500, Jackson), counterstained with DAPI (Sigma-Aldrich), and observed under a fluorescence microscope (BX41TB, Olympus, Japan). Additionally, part of the skin from the same site was fixed in 4% paraformaldehyde, HE stained, and observed for the skin structure and growth of the skin appendages under the optical microscope.

### 2.6. Statistical Treatment

Statistical analyses were performed using SPSS15.0. Measurement data were expressed as *X* ± *S*. Intergroup comparison was performed using *t*-test. *P* < 0.05 was considered statistically significant.

## 3. Results

### 3.1. Evaluation of Cultured ADMSCs In Vitro

**Figure 1:**
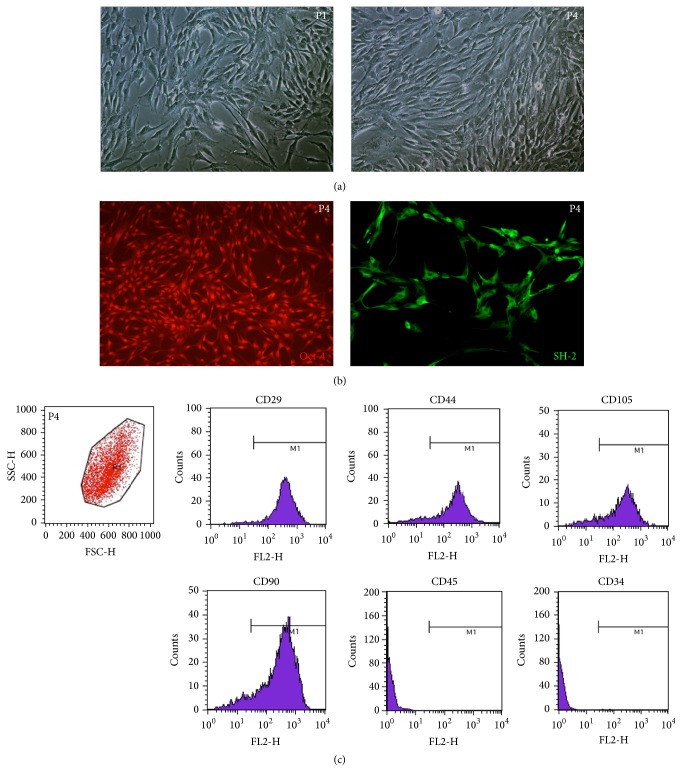
Morphology and molecular marker detection of isolated ADMSCs. (a) Phase-contrast micrographs of ADMSCs at P1 and P4. Most ADMSCs were spindle-shaped with scant cytoplasm and with granules around the nuclei (original magnification 100x). (b) Immunofluorescence detection: P4 ADMSCs were positive for Oct-4 and SH-2 (original magnification 100x). (c) FACS analysis: P4 ADMSCs were positive for CD29, CD44, CD105, and CD90 and negative for CD45 and CD34.

Primarily cultured ADMSCs were spindle-shaped, with a relatively large nucleus-cytoplasm ratio. Confluent cells began forming clones at day 4 after seeding. The size of the clonal cells was relatively small, with a large nucleus-cytoplasm ratio, where split kernels were visible. About 80% of the ADMSCs became confluent 12 days after seeding (Figure 1(a) P1). P4 ADMSCs were morphologically uniform (Figure 1(a) P4). Immunofluorescence staining of the fourth-passage adipose stem cells showed strong positive expression for Oct-4 and SH-2, with a positive rate of more than 90% (Figure 1(b)). Flow cytometry showed that P4 ADMSCs were positive for CD29, CD44, CD105, and CD90 and did not express CD45 or CD34 (markers for hematopoietic stem cells) (Figure 1(c)).

### 3.2. Evaluation of hAM and ADMSCs Seeded on AAM

**Figure 2:**
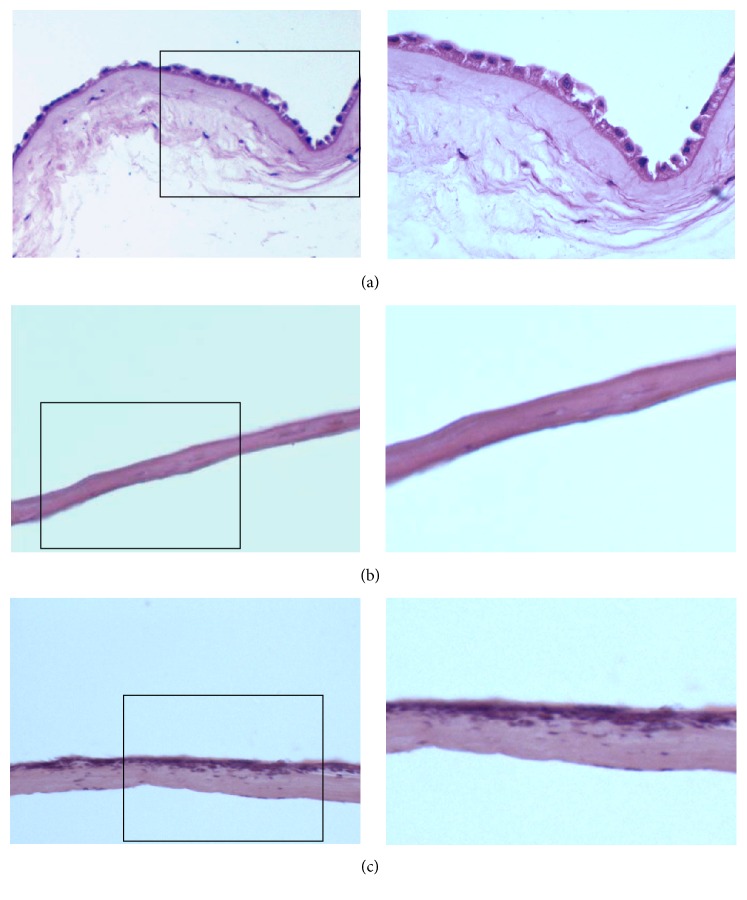
Morphology of hAM (a), AAM (b), and AAM loaded with ADMSCs (c). The right is the magnification of the left. HE staining showed that the epidermal layer of the intact AM was complete and continuous with the nucleus clearly seen and disappeared in AAM. After loading ADMSCs onto AAM, cells covered the surface of AAM and fused into patches.

Optical microscopic observation by HE staining: The epidermal layer of the cryopreserved intact hAM was complete and continuous with the nucleus clearly seen (Figure 2(a)) and disappeared after trypsin treatment but the basement membrane kept intact (Figure 2(b)). At day 3 of loading ADMSCs onto AAM, HE staining of optical microscopy showed that cells grew well with large cell bodies protuberating like spindles. At day 7, cells fused into patches covering the surface of AAM cells and turned from a single layer to multiple layers (Figure 2(c)).

### 3.3. Wound Healing in Different Groups

The healed skin was lower than the normal skin in all groups. At day 7 after seeding, AAM was attached onto the wound surface securely, and the wound surface began shrinking; at day 14 after seeding, AAM fell off from the wound surface and the sound surface further shrank and healed; and at day 28, the wound surface was stabilized. Comparison of the wound healing rate at days 7, 14, and 28 after transplantation showed that the wound healing rate in ADMSC-AAM seeding group was significantly higher than that in hAM and AAM groups, and the wound healing rate in AAM group was significantly higher than that in hAM group (Table 1).

### 3.4. Characteristics of the New Skin Tissue Structure

**Figure 3:**
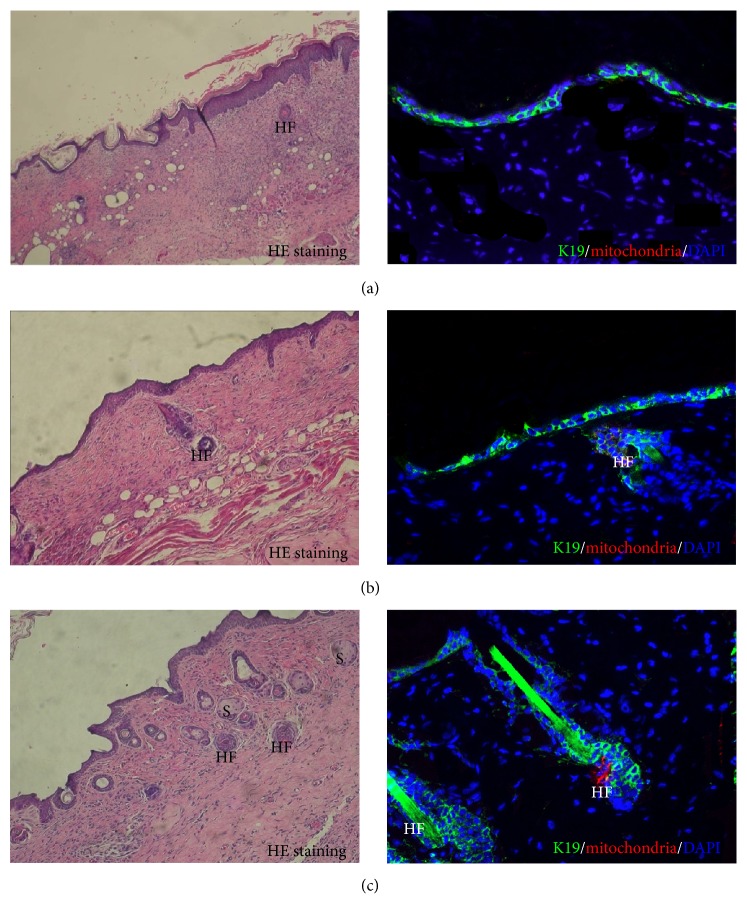
HE staining showed that, at day 28, a lot of typical hair follicle-like structures appeared in ADMSC-AAM seeding group (c) compared with hAM group (a) and AAM group (b). Immunohistochemistry showed that the healed epidermis was positive for cytokeratin 19. Some cells of new hair follicles were positive for both keratin 19 (green) and human derived mitochondria (red). S: sebaceous; HF: hair follicle.

**Figure 4:**
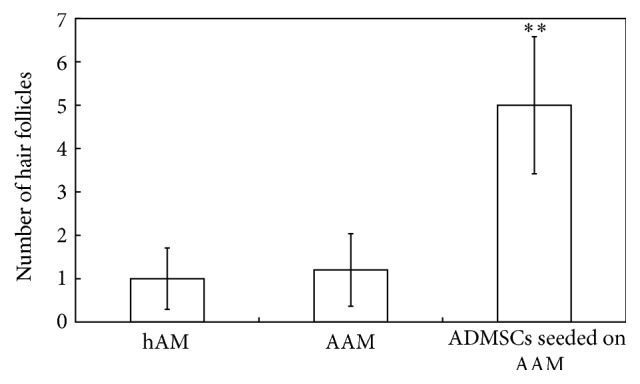
Comparison of hair follicles in different groups, ^*∗∗*^ *P* < 0.05. Image pro plus showed that the number of hair follicles in ADMSC-AAM seeding group was significantly higher than that in hAM and AAM groups.

HE staining showed that, at day 28, the hair follicle-like structure appeared in ADMSC-AAM seeding group; the number of epidermal layers in ADMSC-AAM seeding group was greater than that in hAM and AAM groups, and all wounds in ADMSC-AAM seeding group healed completely (Figure 3). The typical hair follicle structure was observed at day 28 day after transplantation, when immunohistochemistry showed that some cells of new hair follicles in the healed epidermis were positive for both keratin 19 and human derived mitochondria. Keratin 19 is a marker when mesenchymal cells are transcribed to epithelial cells, and anti-human-derived mitochondria are often used as a tracing agent to demonstrate cells derived from human ADSCs. Image pro plus showed that the number of hair follicles in ADMSC-AAM seeding group was significantly higher than that in hAM and AAM groups. The process of hair follicle development showed that composite AAM and ADMSCs not only promoted the healing of the full-thickness defects in the mice but also facilitated generation of the appendages of the affected skin, thus promoting restoration of the skin function (Figure 4). HE staining showed that regeneration of skin appendages, especially regeneration of the hair follicle, is very similar to hair follicle formation in the process of normal skin development.

## 4. Discussion

The skin is known as the largest organ of the human integumentary system and is composed of complex tissue structures including hair follicles, sweat glands, sebaceous glands, and other appendages [6]. It plays important roles in barrier protection, thermoregulation, hair generation, and other physiological functions. The skin covers the body externally, making it vulnerable to injuries from various external factors such as burn, trauma, and chronic ulceration. There are about one million burn patients in China who need skin transplantation annually. However, as dermagrafts currently used in clinical practice contain the epidermis alone or epidermis with dermis but without hair follicles, sweat glands, melanin cells, capillaries, fat layers, and other appendages, they lack many physiological functions of the normal skin, thus seriously affecting the patient's quality of life.

Since the successful isolation of ADMSCs in vitro, they have become the most extensively used adult stem cells in tissue engineering and regenerative medicine [12–14]. ADMSCs express the mesenchymal and pluripotent markers [15, 16] as we examined, and compared with stem cells derived from other sources, ADMSCs have unique advantages of abundant sources, easy obtainment, high proliferative activity, and multidirectional differentiation potentiality and therefore have become common seed cells in tissue engineering [17, 18].

The use of ADMSCs with an AAM coculture system has been reposted as a stem cell therapy and scaffold transplantation for the treatment and functional repair of skin [16, 19]. Sánchez-Sánchez et al. [19] reported that the radiosterilized hAM and pig skin may prove to be suitable scaffolds for delivery of hADMSCs to promote tissue regeneration in skin injuries. Our present study also demonstrated the feasibility of seeding ADMSC onto AAM for the clinical treatment of skin defects. Both ADMSCs and AAM are easily accessible. Unlike fresh hAM, AAM can be preserved for a relatively long time as a safe and effective scaffold material [20]. Composite culture and transplantation of AAM are relatively easy without special technical requirements. The proliferation and differentiation abilities of hAM epithelial cells are not as good as those of ADMSCs. In addition, ADMSCs can differentiate to skin-related tissues [21]. It was found in the present study that the adipose stem cells were able to closely attach to the surface of AAM and grew well 24 h after seeding. At day 7 after seeding, cells fused to patches and covered the surface of the AAM. But as AAM is easily dried, contraction was observed in 50% of the wound area at day 7 after seeding and also in more than one-third of the wound area in ADMSC-AAM seeding group at day 28 after treatment. If this problem could be solved successfully, AAM's clinical value in skin tissue engineering would be further upgraded.

More importantly, we noticed a phenomenon of hair follicle development during the process of using composite ADMSCs and AAM to repair the full-thickness skin defects. Generally, only simple wound coverage is implemented in the process of repairing full-thickness skin defects or deep second-degree burns, without realizing the regeneration of skin appendages and functional recovery. In the present study, we rehearsed the process of skin appendage development and achieved functional recovery of the wounded skin. This result may prove to be clinically significant in the treatment of full-thickness skin defects using skin tissue engineering.

## Figures and Tables

**Table 1:** Comparison of the wound healing rate between different groups.

Groups	Day 7	Day 14	Day 28
hAM	33.39 ± 4.7	43.01 ± 2.8	85.42 ± 1.9
AAM	52.68 ± 3.5^*∗∗*^	68.43 ± 2.4^*∗∗*^	79.48 ± 1.6^*∗∗*^
ADMSCs seeded on AAM	64.38 ± 3.9^*∗∗*^	79.75 ± 3.8^*∗∗*^	92.02 ± 4.2^*∗∗*^

^*∗∗*^ *P* < 0.05.
